# Aquilarin A, a New Benzenoid Derivative from the Fresh Stem of *Aquilaria sinensis*

**DOI:** 10.3390/molecules15064011

**Published:** 2010-06-01

**Authors:** Qing-Huang Wang, Ke Peng, Le-He Tan, Hao-Fu Dai

**Affiliations:** 1 National Center of Important Tropical Crops Engineering and Technology Research, Chinese Academy of Tropical Agricultural Sciences, Danzhou 571737, Hainan, China; E-Mails: wqh3687@163.com (Q.-H.W.); tlh3687@163.com (L.-H.T.); 2 Key Laboratory of Tropical Crop Biotechnology, Ministry of Agriculture, Institute of Tropical Bioscience and Biotechnology, Chinese Academy of Tropical Agricultural Sciences, Haikou 571101, Hainan, China; E-Mail: pengkeke2004@126.com (K.P.)

**Keywords:** *Aquilaria sinensis* (Lour.) Gilg, aquilarinA, cytotoxic activity

## Abstract

Chemical investigation of the EtOH extract of the fresh stem of *Aquilaria sinensis *collected in Hainan Province of China resulted in the isolation of a new benzenoid, named aquilarin A (**1**), together with two known compounds balanophonin (**2**) and (+)-lariciresinol (**3**). Their structures were elucidated by a study of their physical and spectral data. Compounds **2 **and **3 **exhibited cytotoxicity against SGC-7901 and SMMC-7721 cell lines.

## 1. Introduction

**Figure 1:**
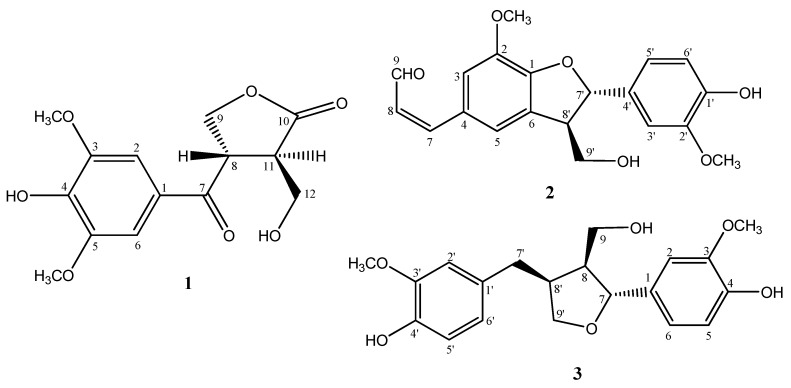
Structures of compounds **1**−**3**.

Agarwood (‘Chenxiang’ in Chinese) is a kind of resinous wood formed by some *Aquilaria* species in response to injury by cutting, holing, burning, or incursion of moths, microorganisms, *etc.*, is well known as incense in the Oriental region, and has also been used as a sedative, analgesic and digestive in Traditional Medicine [1]. Up to now, the formation process of agarwood in trees has not been understood in detail. Comparison of the chemical constituents of the damaged wood with those of the healthy wood is necessary to discover the bioorganic process of agarwood formation. *Aquilaria sinensis (Lour.) *Gilg is the only plant resource in China for agarwood, which is also called Chinese eaglewood, to distinguish it from agarwood of other species, such as *A. agallocha *or *A. malaccensis*. Previous phytochemical investigation on Chinese eaglewood revealed characteristic sesquiterpenes and chromone derivatives [1,2,3,4,5,6], but little is known about the chemical constituents of the healthy wood. In the present paper, we describe the isolation and structure elucidation of a new benzenoid derivative aquilarin A (**1**), together with two known compounds, balanophonin (**2**) and (+)-lariciresinol (**3**) (Figure 1) from the 95 % ethanol extract of the fresh stem of *A*. *sinensis*. Compounds **2 **and **3 **showed growth-inhibitory activity on SGC-7901 and SMMC-7721 cell lines.

## 2. Results and Discussion

**Figure 2:**
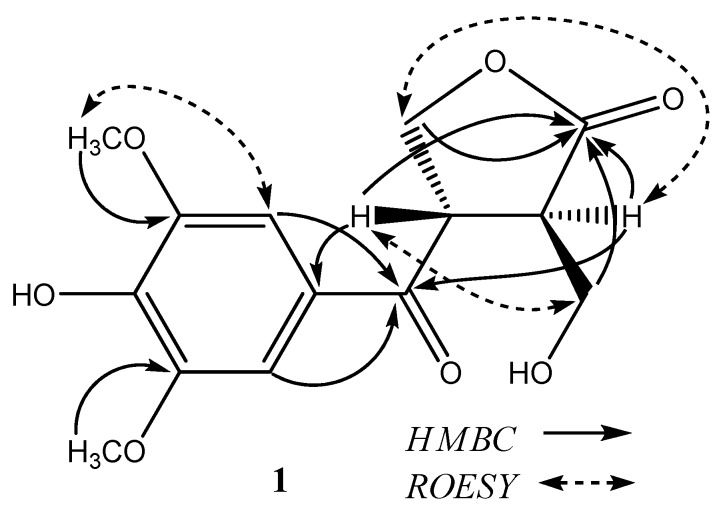
Key HMBC and ROESY correlations of compound **1**.

Compound **1**, was obtained as an amorphous powder. Its HR-ESI-MS spectrum showed the quasi-molecular [M+Na]^+ ^ion peak at *m*/*z *319.0789 (calc. 319.0794), corresponding to the molecular formula C_14_H_16_O_7_. This formula can also be validated through its ^1^H-NMR, ^13^C-NMR and DEPT data. The IR spectrum displayed free hydroxyl (3,428 cm^−1^), γ-lactone carbonyl (1,766 cm^−1^), and aromatic ring (1,586, 1,511 cm^−1^) absorptions. The ^13^C-NMR spectrum of compound **1** (Table 1) revealed two oxygenated methylenes (*δ_C _*58.9 and 68.0), two methines (*δ_C _*43.6 and 45.7), two methoxyls (*δ_C _*56.2 and 56.2), two carbonyls (*δ_C _*195.7 and 176.6), and six aromatic carbons of a symmetrical benzene ring (*δ_C _*125.7, 106.6, 106.6, 147.8, 147.8 and 141.8). The ^1^H-NMR spectrum of **1** (Table 1) showed two singlet aromatic protons at *δ_H_*7.30 (2H, s), two aromatic OMe at *δ_H_*3.84 (6H, s), and one phenolic OH at *δ_H_* 5.29. The remaining oxymethylene [*δ_H_*4.58 (1H, overlapped, H-9α) and *δ_H_*4.20 (1H, dd, *J* = 6.3, 7.7 Hz, H-9β)], two methines [*δ_H_*4.58 (1H, overlapped, H-8) and *δ_H_*3.00 (1H, m, H-11)], and a hydroxymethyl [*δ_H_*3.83 (1H, dd, *J* = 3.5, 11.0 Hz, H-12α), 3.61 (1H, dd, *J* = 3.5, 11.0 Hz, H-12β)] were ascribed to a ^9^CH_2_O−^8^CH−^11^CH−^12^CH_2_OH fragment by ^1^H-^1^H COSY spectrum. The HMBC cross peaks (Figure 2) from the aromatic protons (H-2 and H-6) to C-7 and H-8 to C-1 suggested that C-8 was connected with C-1 through a carbonyl group [*δ_C_* 195.7 (C-7)], and two aromatic OMe should be located at C-3 and C-5 in the symmetrical benzene ring. A γ-butyrolactone ring was deduced from the HMBC cross peaks from H-8, H-9, H-11, and H-12 to the lactone carbonyl [*δ_C _*176.6 (C-10)]. The ROESY correlations from H-8 to H-12 and H-9 to H-11 indicated the *trans* configuration at C-8 and C-11 (Figure 2). On the basis of the above results, the structure of compound **1 **was thus elucidated and named aquilarin A.

**Table 1:** ^1^H- and ^13^C-NMR data of **1 **in DMSO-*d_6_*.(^1^H at 400 and ^13^C at 100 MHz; *J *inHz).

Position	*δ*_C_	*δ*_H_
1	125.7	
2	106.6	7.30 (1H, s)
3	147.8	
4	141.8	
5	147.8	
6	106.6	7.30 (1H, s)
7	195.7	
8	43.6	4.58 (1H, overlapped)
9	68.0	4.58 (1H, overlapped), 4.20 (1H, dd, 6.3, 7.7 Hz)
10	176.6	
11	45.7	3.00 (1H, m)
12	58.9	3.83 (1H, dd, 3.5, 11.0), 3.61 (1H, dd, 3.5, 11.0 Hz)
OCH_3_	56.2	3.84 (6H, s)

Based on the comparison of the ^1^H- and ^13^C-NMR spectral data of compounds **2 **and **3** with those reported in the literature [7,8], compounds **2 **and **3 **were identified as balanophonin and (+)-lariciresinol, respectively.

Compounds **1**−**3** were evaluated for their cytotoxic activity against SGC-7901 and SMMC-7721 cell lines using the MTT method. Compound **2 **showed cytotoxic activity against SGC-7901 cell line, and compound **3 **showed cytotoxic activity against SGC-7901 and SMMC-7721 cell lines, while compound **1** was inactive (IC_50_ > 100 μg·mL^-1^) (Table 2).

**Table 2:** *In vitro* cytotoxicities of compounds **1**−**3 ** (IC_50_ values, *µ*g·mL^-1^).

Compound	SGC-7901	SMMC-7721
**1**	−	−
**2**	34.0	−
**3**	80.0	75.2
Mitomycin C*^a^*	8.8	2.2

*^a^* Positive control.

## 3. Experimental

### 3.1. General

Melting points were obtained on a Beijing Taike X-5 stage apparatus and are uncorrected. Optical rotation was recorded using a Rudolph Autopol III polarimeter (Rodolph Research Analytical, New Jersey, USA). The UV spectra were measured on a Shimadzu UV-2550 spectrometer. The IR spectra were obtained on a Nicolet 380 FT-IR instrument, as KBr pellets. The NMR spectra were recorded on a Bruker AV-400 spectrometer, using TMS as an internal standard. The HRESIMS spectra were measured with an API QSTAR Pulsar mass spectrometer. Column chromatography was performed with silica gel (Marine Chemical Industry Factory, Qingdao, China) and Sephadex LH-20 (Merck). TLC was preformed with silica gel GF254 (Marine Chemical Industry Factory, Qingdao, China).

### 3.2. Plant material

Fresh stems of *A*. *sinensis *(Lour.) Gilg were collected in Ding’an county, Hainan province, China in November 2008, the plant was identified by Associate Professor Zheng-Fu Dai of the Institute of Tropical Bioscience and Biotechnology, Chinese Academy of Tropical Agricultural Sciences, where a voucher specimen (No. AS20081101) was deposited.

### 3.3. Extraction and isolation

The fresh and crushed stems of *A*. *sinensis *(66.0 kg) were extracted with 95% EtOH three times (100 L × 3) at room temperature. After removal of EtOH by evaporation, the EtOH extract was suspended in water (10.0 L) and successively partitioned with petroleum ether, EtOAc, and then *n*-BuOH to give the corresponding Petro-extract (106.3 g), EtOAc-extract (66.0 g), and *n*-BuOH-extract (244.5 g), respectively. 

The EtOAc fraction (66.0 g) was subjected to vacuum liquid chromatography (VLC) over silica gel, eluting with a gradient of CHCl_3_-MeOH (1:0**−**0:1, v/v) to afford eight fractions (Fr.1**−**8). Fr.2 (25.0 g) was chromatographed on a silica gel column using a step gradient elution of Pet-Acetone (1:0**−**0:1, v/v) to afford eight fractions (Fr.2-1**−**8). Fr.2-4 (6.5 g) was subjected to column chromatography over Sephadex LH-20 using CHCl_3_-MeOH (1:1, v/v) as eluent to afford four fractions (Fr.2-4-1**−**4). Fr.2-4-1 (554.0 mg) was separated by column chromatography over Sephadex LH-20 using CHCl_3_-MeOH (1:1, v/v) as eluent to afford compound **1** (5.0 mg). Fr.2-4-2 (4.4 g) was submitted to column chromatography over silica gel, eluenting with gradient CHCl_3_-MeOH to afford compounds **2 **(10.0 mg) and **3 **(3.0 mg).

### 3.4. Characterization of Compounds ***1−3***

*Aquilarin A *(**1**): Armorphous powder, M.p. 167−168 ºC. [*α*]^25^_D_ = − 78.3 (*c *= 0.6, MeOH). UV (MeOH): λ_max _(log *ε*_max_): 306 (1.41), 226 (1.66), 214 (1.39) nm. IR (KBr): ν = 3,428, 2,920, 2,851, 1,766, 1,586, 1,511, 1,464, 1,380, 1,116 cm^-1^. HR-MS [(+)-ESI]: *m/z* = 319.0789 (calcd. 319.0794 for C_14_H_16_O_7_Na, [M + Na]^+^). ^1^H and ^13^C-NMR: see Table 1. 

*Balanophonin* (**2**): Yellow oil, [*α*]^25^_D_ = + 12.1 (*c *= 1.0, CHCl_3_). ESI-MS *m/z*: 379 [M+Na]^+^. ^1^H-NMR (400 MHz, CDCl_3_): *δ* 9.62 (1H, d, *J *= 7.8 Hz, H-9), 7.41 (1H, d, *J *= 15.8 Hz, H-7), 7.13 (1H, d, *J *= 1.5 Hz, H-5), 7.03 (1H, d, *J *= 1.5 Hz, H-3), 6.90 (1H, d, *J *= 1.6 Hz, H-3'), 6.89 (1H, d, *J *= 8.0 Hz, H-5'), 6.88 (1H, d, *J *= 8.0 Hz, H-6'), 6.59 (1H, dd, *J *= 7.7, 15.8 Hz, H-8), 5.63 (1H, d, *J *= 7.1 Hz, H-7'), 3.97 (2H, m, H-9'), 3.67 (1H, dd, *J* = m, H-8'), 3.92 (3H, s, 2-OCH_3_), 3.86 (3H, s, 2'-OCH_3_). ^13^C-NMR (100 M Hz, CDCl3): *δ* 151.5 (C-1), 144.8 (C-2), 112.2 (C-3), 128.1 (C-4), 118.2 (C-5), 129.1 (C-6), 153.2 (C-7), 126.3 (C-8), 193.6 (C-9), 145.9 (C-1'), 146.7 (C-2'), 108.7 (C-3'), 132.2 (C-4'), 119.4 (C-5'), 114.4 (C-6'), 88.9 (C-7'), 53.0 (C-8'), 63.9 (C-9'), 56.0 (2×OCH_3_).

*( + )-Lariciresinol *(**3**): Armorphous powder. [*α*]^25^_D_ = + 31.0 (*c *= 0.5, MeOH). UV (MeOH): λ_max _(log *ε*_max_): 221 (1.02), 282 (2.17) nm. ESI-MS *m/z*: 383 [M+Na]^+^. ^1^H-NMR (400 MHz, CDCl_3_): *δ* 6.88 (1H, d, *J *= 1.8 Hz, H-2), 6.87 (1H, d, *J *= 8.0 Hz, H-5), 6.84 (1H, d, *J *= 8.0 Hz, H-5'), 6.81 (1H, dd, *J *= 1.8, 8.0 Hz, H-6), 6.70 (1H, dd, *J *= 1.8, 8.0 Hz, H-6'), 6.69 (1H, d, *J *= 1.8 Hz, H-2'), 4.79 (1H, d, *J *= 6.6 Hz, H-7), 4.06 (1H, dd, *J *= 6.6, 8.6 Hz, H-9'a), 3.92 (1H, dd, *J *= 8.1, 10.9 Hz, H-9a), 3.89 (3H, s, OCH_3_), 3.88 (3H, s, OCH_3_), 3.79 (1H, dd, *J *= 6.5, 10.8 Hz, H-9'b), 3.75 (1H, dd, *J *= 6.6, 8.6 Hz, H-9b), 2.92 (1H, dd, *J *= 5.2, 13.5 Hz, H-7'a), 2.73 (1H, m, H-8'), 2.55 (1H, dd, *J* = 10.7, 13.5 Hz, H-7'b), 2.41 (1H, m, H-8).^ 13^C-NMR (100 MHz, CDCl3): *δ* 134.8 (C-1), 108.3 (C-2), 146.5 (C-3), 145.1 (C-4), 114.2 (C-5), 118.8 (C-6), 82.9 (C-7), 52.6 (C-8), 61.0 (C-9), 132.3 (C-1'), 111.2 (C-2'), 146.6 (C-3'), 144.0 (C-4'), 114.4 (C-5'), 121.2 (C-6'), 33.4 (C-7'), 42.4 (C-8'), 72.9 (C-9'), 56.0 (2×OCH_3_).

### 3.5. Bioassay

The 3-(4,5-dimethylthiazol-2-yl)-2,5-diphenyltetrazolium bromide (MTT) assay was performed according to the previously reported method [9]. The inhibition rates (IR%) were calculated using OD mean values from IR% = (OD_control_ − OD_sample_)/OD_control_. The IC_50_ value, which is defined as the concentration of sample needed to reduce 50% of absorbance relative to the vehicle-treated control, was determined using the Bliss method. The same experiment was repeated independently three times to obtain a mean IC_50_ value and its standard deviation. The IC_50_ values are listed in Table 2.

## 4. Conclusions

Although much attention has been paid to the phytochemical investigation of Chinese eaglewood, little is known about the chemical constituents of the fresh healthy wood. Previous studies revealed the characteristic components of Chinese eaglewood were sesquiterpenes and chromone derivatives [1,2,3,4,5,6]. In our present study a new benzenoid derivative, aquilarin A (**1**), together with two known lingnans balanophonin (**2**) and (+)-lariciresinol (**3**), were isolated from the 95% ethanol extract of the fresh stem of *A*. *sinensis*, which were different from those of Chinese eaglewood. Meanwhile, the cytotoxicity against SGC-7901 and SMMC-7721 cell lines of compounds **1**− **3** was evaluated for the first time.
